# Scaling up of parenting support to prevent violence against children in Tanzania: insights from policymakers and service providers

**DOI:** 10.1186/s43058-024-00684-8

**Published:** 2025-01-13

**Authors:** Sabine van Tuyll van Serooskereken Rakotomalala, Kija Nyalali, Joyce Wamoyi, Onduru Gervas Onduru, Gerry Mshana, F. Marijn Stok, Mara A. Yerkes, John B. F. De Wit

**Affiliations:** 1 Department of Interdisciplinary Social Science, Utrecht University, Utrecht, Netherlands; 2 Department of Sexual and Reproductive Health, National Institute for Medical Research, Mwanza, Tanzania; 3 Ministry of Health, National TB and Leprosy Program, Dodoma, Tanzania; 4 Sexual and Reproductive Health Programme, National Institute for Medical Research, Mwanza, Tanzania

**Keywords:** Violence against children, Parenting support, Implementation, Scale-up, Policy, Policymakers, Service providers

## Abstract

**Background:**

Evidence shows that parenting behaviours, including the use of violent discipline, can be changed through programmatic interventions. This study seeks to examine how policymakers and service providers in Tanzania perceive the provision of parenting support as a strategy to prevent violence against children and what the enabling and hindering factors are for the scale-up of existing evidence-based parenting supports. It does this by applying Daly’s analytical framework for parenting support.

**Methods:**

Qualitative research was undertaken, with interviews conducted with 20 key informants consisting of purposively sampled policymakers and service providers. The interview data were analysed using inductive and deductive coding and analysis.

**Results:**

The most prominent enabling factors noted for the scale-up of parenting support interventions in Tanzania include the existing supportive political commitment, the interventions currently on offer at the programmatic level, and the perceived understanding of Tanzanian caregivers of the importance of parenting and, thereby, a willingness to change. Current factors hindering the scale-up include the lack of a common understanding of what evidence-based parenting programmes entail, inadequate provision of human and financial capital to implement the programmes using community resources and deeply engrained social norms around adultism and gender.

**Conclusion:**

Daly’s analytical framework allowed us to examine barriers and facilitators to scale-up the provision of parenting support to prevent violence against children, based on the viewpoints of policymakers and service providers. Understanding these barriers and facilitators will allow Tanzanian policymakers and service providers to further close the gap between the policies and the actual implementation of evidence-based parenting support programmes aimed at preventing violence against children.

**Supplementary Information:**

The online version contains supplementary material available at 10.1186/s43058-024-00684-8.

Contributions to the literature

- While parenting support is an effective strategy to reduce parental violence, implementation at scale in countries around the world is limited.
- The current study contributes in-depth insight into policymakers' and service providers' views on why coverage of parenting support is limited in Tanzania, an understudied region.
- According to informants, factors enabling parenting support in Tanzania include policy and programmatic support and parents' willingness to change at a societal level.
- Factors noted that hinder implementation of parenting support in Tanzania include engrained norms around adultism and gender, and limited available human and financial resources at community levels.

## Background

Globally, it is estimated that up to 1 billion children aged 2–17 years, that is, one out of two children worldwide, have experienced physical, sexual, or emotional violence or neglect in the past year [1]. Violence against children has lifelong impacts on the health and well-being of children, families, communities, and nations. Corporal punishment, for example, negatively impacts the mental and physical health of children in the short- and long-term as well as their level of educational attainment [2, 3]. Irrespective of degree or intensity, any form of violence against children carries an inbuilt risk of escalation and, thereby, a heightened risk of parents perpetrating severe maltreatment of their offspring [4]. Research shows that ‘eradicating maltreatment’ during childhood would reduce the overall rate of depression in adulthood by more than half, alcoholism by two-thirds, and suicide, serious drug abuse, and domestic violence by three-quarters [5].

As a key strategy to prevent violence against children, the past 20 years have seen a steady increase in the development, implementation, and evaluation of evidence-based parenting support (further referred to as EBPS) [6]. EBPS refers to the full breadth of empirically supported means of helping parents raise their children, ranging from internet-based and home visitation programmes to group and individual face-to-face delivery of parenting interventions [7]. For the purpose of this article, the terms “programmes” and “interventions” are used interchangeably. EBPS positively influences parenting beliefs and behaviours, including close monitoring and more frequent positive parent-child interactions [8]. In addition to being a key strategy for the prevention of family violence, EBPS also positively affects a range of other child and parent outcomes, including child and adolescent mental and physical health; child and adolescent academic attainment; parental well-being and mental health; and parental skills, knowledge, and confidence [9]. Therefore, the quality of parenting and family life is often considered ‘the most important of all the potentially modifiable influences affecting children’s development and mental health across the life course’ [10].

Despite compelling evidence from randomized trials in high-, middle- and low-income countries, addressing violence against children remains a low priority for many governments [11]. Consequently, the availability of EBPS globally is limited, with only 25% of countries worldwide providing support to parents who need it [12]. In Australia, one of the 25% mentioned above of countries where parenting support is being provided, the coverage is limited, with only 35% of parents who have a child needing help for a behavioral problem report having their needs fully met [13]. In sum, violence against children is a major public health issue worldwide, and the provision of EBPS can address it, yet many countries do not implement these interventions at scale.

Research has been conducted on how parenting behaviours can be changed through interventions [14], how EBPS can be scaled up [15], and how behavioural science can be integrated into government policies [16, 17]. However, the behaviours and decisions of policymakers and service providers that may facilitate the widespread implementation of EBPS are yet to be understood [18]. Moreover, knowledge of EBPS remains slightly dominated by Western research, which is problematic, since attitudes and behaviours around parenting support are deeply ingrained in cultural and societal values [19].

Tanzania is a country that has high rates of violence against children and has put parenting support high on the political agenda. In Tanzania, 56.5% of girls and 52.7% of boys experience some form of physical violence (including being punched, kicked, whipped, beaten with an object; choked, smothered, attempted to drown, burned; and/or threatened/victimised with a weapon); and approximately 26.7% of girls and 11.6% of boys are exposed to sexual violence before reaching the age of 18 [20]. A study conducted with 700 Tanzanian secondary school students demonstrates that more than 90% of all students reported exposure to violent discipline by a parent within the past year, and more than 80% of parents acknowledged using violent discipline techniques due to parental stress [21].

Despite these high rates of violence, the political will in Tanzania to provide parenting support is evident. Ministerial jurisdiction for parenting support varies from country to country, including health, social welfare, justice, and education sectors [22]. In Tanzania, parenting support falls under the Ministry of Community Development, Gender, Women, and Special Groups, which is responsible for policies and programmes concerning family welfare, child development, women's empowerment, and vulnerable groups. The ministry plays a key role in creating and implementing policies related to parenting education, child protection, and family support. Additionally, the Ministry of Health has a role in early childhood development, maternal and child health, and nutrition, indirectly impacting parenting support. Policies that mention the importance of supporting parents include, for example, the 2021–2026 Tanzania National Development Plan, the National Agenda for Responsible Parenting and Family Care (publication forthcoming), and the Five-year National Plan of Action to End Violence Against Women and Children 2017/18–2021/22. While policies are available, a scoping review conducted by the authors (not included for space reasons but available upon request) indicated a dearth of academic research on their effective implementation.

### This study

The current study is the first of its kind for Tanzania. It aims to unpack how policymakers and service providers view potentially contentious parenting practices, such as violent disciplining, as well as the provision of parenting support as a strategy to prevent violence against children. In doing so, it seeks to explore the factors enabling and hindering the scale-up of existing EPBS. The study contributes to the child and adolescent health field by assessing the value of parenting support as a strategy to prevent violence against children [23] and to implementation science by examining the key factors that enable or hinder the implementation and scale-up of EBPS [10]. The study also contributes to the Parenting for Lifelong Health Scale-Up of Parenting Evaluation Research (SUPER) Study, thereby growing the evidence base that can inform the scale-up of EBPS through policies and practice [15].

Daly’s [24] analytical framework for parenting support (further referred to as Daly’s framework; see Table 1) provides a valuable analytical perspective for analysing the enablers and barriers around the provisions of EBPS in society and politics. The framework recognizes the complexity of parenting support policies, which include both an element of being a social policy (i.e., an institutionalized response to collective problems) [25] as well as being an active social and political intervention in the lives of parents [24].

**Table 1:** Daly’s analytical framework for parenting support [24]

Social Policy Form	What is the content or core offer?
	What are the modalities?
	Where is parenting support lodged?
	How does it develop over time and what are the historical roots?
	What are the philosophical and progressional influences?
Social and Political Intervention	What or who are the key actors promoting parenting support?
	What are the main claims or rationales for parenting support in relation to a) children, b) parents and c) families and society?

Acknowledging that policies and interventions are normative and thus value-laden [26], Daly’s framework starts by considering the offer or ‘good’ made available to parents and the modality of provision. It then considers the provision's location in the policy landscape and its linkages to other policies, historical roots and philosophical underpinnings, and dominant professional influences. These aspects matter because they help question to what extent parenting support should focus on babies and toddlers versus supporting older children; to what extent developments in parenting support are demand or supply-led; and to what extent parenting support may be a foreign concept being imported into Tanzania. The second aspect, the social and political interventions, can be investigated by identifying the main actors involved in parenting support and the rationales taken forward by parenting support policy. Whether parenting support is considered a health, social services, income, or justice issue depends on which group of actors is involved. Moreover, understanding the main rationales behind the promotion of parenting support is essential to understanding which policymaker-defined problem parenting support is trying to address in Tanzania. This study extends Daly's work by applying her framework to Tanzania. The Sub-Saharan Africa region is not often included in research on parenting support. By focusing on this understudied region, the authors foreground the perspectives of policymakers and service providers to understand what may facilitate or impede the effective scale-up of EBPS in Tanzania.

## Methods

The Ethics Review Board of the Faculty of Social & Behavioural Sciences at the University of Utrecht approved this study (Protocol 23–0063). The protocol was also approved by the Tanzanian National Medical Research Council (NIMR/HQ/R.8a/Vol.IX/4218) as part of a larger trial of a digital parenting intervention to prevent violence against children (i.e., ParentApp-teens) with a component on policy engagement [27].

Purposive sampling was used to carefully recruit informants to yield an in-depth understanding of this topic. Inclusion criteria for policymakers were twofold: 1) a policymaker or government official whose work involves decision-making on interventions for families with young children in Tanzania; or 2) an individual who oversees parenting policies and programmes and who has knowledge of parenting programmes and or other related social programmes in the country. Inclusion criteria for personnel in service-providing agencies were any professional, coordinator, or director, working for an evidence-based parenting intervention/programme, currently or in the past. This includes UN agencies, bilateral agencies (such as the CDC), and non-governmental organizations that often support the service-providing agencies.

Fieldwork was conducted in Tanzania in May 2023, by two interviewers from the Tanzanian National Institute for Medical Research (NIMR) and the University of Utrecht. Informants were invited through an email invitation letter by NIMR staff. The interviewers conducted twelve semi-structured Key Informant Interviews (KIIs) and three Focus Group Discussions (FGD), engaging 20 informants: seven central government policymakers (PM) and 13 service-providing (SP) agencies. While the initial plan was to organize only FGDs, this proved logistically complex as informants were located between Dar-Es-Salaam and Dodoma. The combination of KIIs and FGDs allowed more informants to participate. All KIIs and FGDs were conducted in English, except one KII in Kiswahili, due to the informant's preference for using the national language. Interviews with the Ministry of Health, the Ministry of Constitution and Legal Affairs, and the University of Dodoma were not conducted, as the individuals were unavailable during the fieldwork period. Table 2 provides an overview of the informants’ organizational affiliations and indicates which format they participated in. Individual characteristics (e.g., gender or age) are not presented to ensure participant anonymity.

**Table 2:** Primary organizational affiliation of informants interviewed in the study (by chronological order of interviews conducted)

Informant	Affiliation	Ministry/Agency	FGD or KII
1.	SP	WHO	FGD 1
2.	SP	WHO	FGD 1
3.	SP	WHO	FGD 1
4.	SP	WHO	FGD 1
5.	SP	Plan International	KII
6.	PM	Ministry of Home Affairs (MoHA)	KII
7.	PM	Institute of Social Work (IoSW)	KII
8.	PM	Ministry of Community Development, Gender, Women and Special Groups (MOCDGWSG)	KII
9.	SP	Tanzania Early Childhood Development Network (TECDEN)	KII
10.	SP	ICS – Skillful Parenting	KII
11.	PM	MOCDGWSG	KII
12.	PM	President's Office, Regional Administration and Local Government Tanzania (PO-RALG)	KII
13.	PM	Ministry of Education, Science and Technology (MoEST)	KII
14.	PM	MOCDGWSG	KII
15.	SP	PACT Tanzania	KII
16.	SP	Msichana Initiative	KII
17.	SP	UNICEF	FGD 2
18.	SP	UNICEF	FGD 2
19.	SP	United States Centers for Disease Control and Prevention	FGD 3
20.	SP	United States Centers for Disease Control and Prevention	FGD 3

An interview guide was developed using Daly’s framework, with questions around policymakers’ and service providers’ views about childrearing and parental violence; the willingness of parents to partake in parenting support programmes; the interventions offered to parents and how they are provided; what the policy landscape looks like; how policies and practice have developed over time; and what else might be needed to scale-up parenting support [24]. Informants were invited to share both their institutional position and their personal views regarding parental support to prevent violence against children [28]. The KIIs and FGDs were voice-recorded and exported for transcription to Amberscript, an online Artificial Intelligence (AI)-based tool with 85% accuracy in English. The transcription translated from Kiswahili to English using AI was not of high enough quality to be included in the review, which reduced the number of informants from 20 to 19. The other transcripts were examined and lightly edited for readability purposes.

Data analysis was undertaken using both inductive and deductive coding [29]. Using Daly’s framework as guidance, the two interviewers reviewed five interviews separately, to identify the main themes. They then agreed on the following codes or organizational categories: Advocacy, Child Participation, Child Rearing, Collaboration, Gender, Financial Resources, Humanitarian, Influences, Intervention, Lodged, Male Engagement, Media, Political Will, Quality, Monitoring, Rationale, Scale, and Trust. Once agreement was reached about the codes, the first author further reviewed the transcripts, coding them using NVIVO 14 qualitative coding software.

## Results

The analysis of interview data revealed four key themes: political commitment and policy environment; interventions on offer to parents and modalities of provision; resources and capacity affecting access; and reasons for offering parenting support interventions.

### Political commitment and policy environment

Informants identified three elements of political willingness to facilitate the implementation of EBPS: the ratification of international and regional conventions and protocols that encourage the provision of parenting support, the dedication of key politicians and policymakers, and the commitment to collect and publish national data on the prevention of violence.

Conventions: “*Maybe progress is because of the international and regional conventions. Adopting those protocols and conventions and ratifying to our Tanzanian context, means that at least we are bringing changes from those other countries.*” [informant 6, MoHA]

Dedication: “*The Minister for Community Development, gender, women and special groups is herself is going from one place to another to speak on the importance of positive parenting. There is now also a national secretariat for the Early Childhood Development (ECD) programme at the national level, and in that secretariat, a nominated agency is leading parenting support. And parliament members are also champions for ECD.*” [informant 1, WHO]

Data: “*The (National Violence Against Children) survey has influenced pretty much all of the child protection interventions. It led, to some extent to the National Action Plan on Violence Against Women and Children, for which parenting is one of the core components."* [informant 17, UNICEF]

Despite this government commitment at the national level, political will has not yet trickled down to the local government level (council and district), and several informants agreed that information on the supply of EBPS is needed to increase demand.

“*There are several initiatives like the National Multi-Sector Early Childhood Development Program which also speak of responsive caregiving. However, we haven't done much to keep parents understanding the importance of responsive caregiving. It's high time now for the government to mainstream parenting programmes to the community and create a demand for parenting programmes from the community.*” [informant 9, TECDEN]

### Interventions and services on offer to parents

At the time of final data collection (May 2023), seven EPBSs to prevent violence against children were being implemented in Tanzania in their original or modified versions. Two of these have not yet published study results (Table 3). These programmes serve as an essential basis for considering scale-up across Tanzania.

**Table 3:** EBPS in Tanzania, May 2023

**Name**	**Aims of the programme**	**Age of children (years)**	**Setting & Delivery Method**	**Number of sessions & duration (months)**	**Study reference**
**The Skillful Parenting and Agribusiness - ICS**	ICS educates local field trainers, who in turn provide training for parents and teachers. For a duration of twelve months, parents get together weekly and, in group context, work on seven different modules on parenting. Parents generate new knowledge, reflect on their role in the upbringing of their child, share their experiences with each other and discuss the daily challenges of childrearing.	0–18	Community / group	12 sessions/ months	[30]
**Whole School Approach (WSA)**	The WSA, implemented by ICS, is a complex intervention that uses an individual and group dynamics behaviour-change strategy to address school-based violence prevention. The WSA's five components include: (1) school leadership support and training, (2) teacher and support staff training and skills development, (3) life skills and values education for learners, (4) parent and caregiver engagement and training and (5) community partnerships and child protection mechanisms.	10–18	School and Community / in person	13 sessions/10 months	[31]
**The Happy Toto Game**	A mobile learning game to prevent child sexual abuse.	6–36 months	Community / digital	not specified	[32]
**Furaha Teens (local name for Parenting for Lifelong Health program)**	To reduce adolescent exposure to violence in the home and community by improving positive parenting and parent-child communication while reducing familial conflict, harsh discipline, parenting stress, adolescent conduct problems, risky behavior, and ill mental health.	9–14	Community / in person	14 sessions / 3 months	[15]
**The Families Matter! Programme (FMP)**	To promote positive parenting and effective parent-child communication about sexuality and sexual abuse risk reduction.	9–12	Community / in person	6 sessions/ 6 months	[33]
**The Faithful House (TFH)**	TFH is a 3-day faith-based skills-building curriculum that aims to increase household resilience by strengthening families. TFH draws on improved communication and conflict resolution skill building and the individual's faith values as a catalyst for transformation in attitudes regarding gender roles in caregiving and the use of violence in the home (study results currently unavailable for Tanzania).				[34]
**Child Survival and Development Programme (CSDP)**	CSDP is a comprehensive programme focused on improving child survival and development outcomes in Tanzania. It includes various components, such as maternal and child health services, nutrition support, early childhood education, and parenting education (study results currently unavailable for Tanzania).				
**Safari ya Malezi**	Also noteworthy is "Safari ya Malezi," a radio programme sponsored by BBC Media that aims to educate parents and caregivers on positive parenting and early childhood development. The programme airs experiences concerning achievements and challenges from various stakeholders and practitioners on positive parenting and family care.				

Informants noted that communications around different programmes' availability is leading to their scale-up.

*"Different companies come up with different programmes. They even advertise them on social media.*” [informant 1, WHO] *"Stakeholders are currently building on existing programmes by exploring digital delivery.”* [informant 11, MOCDGWSG]

Parenting support programmes generally include modules such as nonviolent discipline, positive reinforcement, parental self-management, and improving parent-child communication.

An important finding that emerged from the interviews was the conceptual confusion around what a parenting programme entails; views ranged from prison visits and nutritional programmes to school feeding programmes and churchgoing to impart parenting skills. On the other hand, representatives from the MOCDGWSG and the frontline service-providing agencies (e.g., ICS, UNICEF, and CDC) could explain their operating model and programme components clearly. Informant 10 [ICS], for example, described the programme as follows:

*"We facilitate parents to do a very critical reflection on how they raise children. Do they think that kind of style is okay with them? What about their values? What about the norms that they practice? Are there different positive ways to raise children?”*

### Resources and capacity for access

The respondents considered four domains key to implementing and scaling up EBPS: capacity building, targeted support, coordination, and research.

Regarding the first point, there was a strong perception by several key informants that capacity building is needed for all stakeholders potentially involved in parenting support programmes [informants 3, 6, 8, 9, 11, 12, and 20].

“*I think that capacity is for everyone because I even had doctors themselves saying that we need that capacity to be able to be good parents. So capacity is for everyone from the national to subnational level, to the ministerial level, policymakers to everyone.”* [informant 3, WHO]

Looking specifically at who should be trained, three informants indicated that front-line health and community development officers, who are the custodians of this work, need to be trained to scale-up programmes across the country intentionally. Taking capacity building a step further, four respondents agreed that funds are not being allocated to building staff capabilities.

“*If we are looking into the programme, one of the constraints would be the budget. You need to have really enough funds to be able to scale it up.”* [informant 15, PACT]

In terms of targeted support, while the government and service-providing agencies are implementing some EBPS, the reach and effectiveness of these parenting support interventions are questioned, particularly when comparing rural and urban areas. The statements below, for example, indicate the need for more visible, targeted, and accessible EBPS.

*“In the rural areas, the women are playing a big role in terms of raising the children. But if you check at the urban level, most of the parents and caregivers are very busy with their work with business, generating income, and doing whatever they can, and the role of taking care of the children remains with the house girls or nannies. When we speak of parenting, we need to understand all these kinds of dynamics."* [informant 5, Plan]

Coordination (i.e., who is doing what and where) of service providers is essential to ensure scale-up and reach. As addressed by informant 12 [PO-RALG]:

*"You first need to understand how the government operates in Tanzania, because the government is a very big machine. And the way it is structured, has an impact even in the coordination matters and in issues of authority, because in Tanzania we have the central government, and we also have the local government…So, we should maybe think of moving it and put it (EBPS) under the Prime Minister's office or the President's office.”*

Lastly, in order to understand which interventions work for which parents, the importance of data and research was raised by informant 8 [ MOCDGWSG] as follows:

*“Sometimes we don't commission research because there are no resources to do that. So, there's a mismatch: because in reality, to determine the rationale for the development of a policy, you should first state clearly, scientifically, that there is a gap."*

### Reasons for participating in parenting support interventions

Parents can access parenting support with different aims, from improving child outcomes (especially in education and health) and preventing child risks (for example, substance abuse and poor mental health) to parental well-being as essential to child well-being. All three themes emerged from the interviews.

Improving child outcomes: "*You have to make sure that you parent well your kids to be a good citizen.”* … “A *paradigm shift is currently taking place whereby parents are getting sensitized to using alternatives to caning.”* [informant 1, WHO]

Preventing child risks: "*Our children are growing without having the skills to navigate the complex environment that they find themselves in. But also, there is so much influence from outside. So basically, parents need help.”* [informant 7, IoSW]

Parental well-being “*The social support structure has changed over time. When my mother was raising us, she had a lot of social support socially. Meaning that we had a lot of people in the house. But now, for me, as I'm raising my children, I don't have that support*”. [informant 3, WHO]

Despite feeling that positive changes are happening, one informant said there is still resistance to parenting without the use of violence.

"*There is probably a small percentage who will be against it, because in every community there's a percentage of people who are radical, and they will not accept anything that they think is going to change them or try to teach them a different way. But that is usually a very small percentage in the normal curve. You'll find them on the extremes, and probably it is in every society. It's just the way that populations are.”* [informant 17, UNICEF]

Another informant expressed that this resistance in Tanzania may be due to the idea that some parents feel that modern parenting is not working for children today.

“*There is a transition between what he would call the old way of parenting that he went through and what is now being referred to as modern parenting. If you ask him, he will opt for the traditional way because modern parenting is not working. It feels as though when you transition into modern parenting, you're losing the grip on your child and can no longer shape his/her behaviours*”. [informant 7, IoSW]

Almost all the informants raised the issue of gender roles. They indicated that women are mainly responsible for child rearing in Tanzania and are, therefore, more likely than men to participate in parenting interventions. Men will join parenting programmes when linked to economic empowerment groups.

“*The analysis shows in every programme around 70% of the attendees are women and not men. So, when we are thinking of the scale-up of parenting interventions in the country, we should also think about how we are going to reach men. As they see the value over time, by increasing their income and improving their parenting skills, they continue to participate in the programmes. Thus participation, of women and men, is not motivated by money. However, men will be more likely to attend if it can be linked to their main duty which is income generation.”* [informant 10, ICS]

## Discussion

This study sought to explore the factors that enable or hinder the implementation of parenting support policies and the expansion of existing EBPS in Tanzania. Research from four sub-Saharan African countries confirms the crucial role of a solid parent-child relationship in preventing and mitigating the effects of adverse childhood experiences, particularly violence against children [35–37]. Additionally, evidence underscores the importance of policies that foster supportive family environments for children to thrive, which is vital in all contexts, including sub-Saharan Africa. However, references to these policy initiatives from the global south, as well as discussions around their implementation challenges, are often missing in parenting support literature [35].

Applying Daly's framework to Tanzania, a sub-Saharan country, the authors conducted a qualitative study with Tanzanian policymakers and service providers to gain a deeper understanding of the process of implementing and scaling up parenting support initiatives. According to policymakers and service providers, the most prominent enabling factors for scaling up parenting support interventions in Tanzania are threefold: the existing political commitment and enabling policy environment, the interventions currently on offer at the programmatic level, the and perceived understanding of the importance of parenting among Tanzania parents and their expected willingness to change.

Policy support for EBPS is mainly established within the Ministry of Community Development, Gender, Women, and Special Groups and the Ministry of Health. This could indicate that in the perception of Tanzania policymakers, while support to parents with babies and toddlers is necessary, this support should be continued throughout childhood. However, according to the policymakers and service providers, to be operative, the policy frameworks would need to describe the components of parenting interventions that need to be in place for the intervention to be effective (e.g., nonviolent discipline techniques, positive reinforcement, and improving the parent-child relationship). While this is done for a small number of the EBPS, this level of detail is missing in the policies and for most interventions. In addition, the modalities of parenting support matter. Currently, parenting support interventions in Tanzania typically consist of group or individual structured sessions conducted by professional or paraprofessional staff, delivered in the home, at a center, or online. While interventions use these different implementation modalities throughout the country, information about their availability is lacking, as is research on the effectiveness of these various modalities.

According to the literature, rationales for partaking in parenting support interventions can be clustered into three categories, namely: 1) for the improvement of child outcomes generally, especially concerning education and health; 2) for the prevention of child risks, specifically domestic violence, substance abuse and poor mental health; and 3) focused on parents and parental well-being as essential to child well-being [24]. All three conceptualizations around the importance of providing EBPS in Tanzania emerged from interviews with the policymakers and service providers, indicating that largely most Tanzanian parents would be willing to partake in EBPS for one reason or another. In sum, according to the informants, if policies, practicalities, and rationales for partaking in parenting support could be clearly expressed and communicated, scale-up and coverage of EPBS would increase.

### Implications for Daly’s framework

It is worth noting that some aspects of Daly’s framework [24] did not feature prominently in participants’ views. These include, firstly, the role that key actors play in promoting EBPS and, secondly, the need to consider the integration of cash transfers with EBPS. Regarding key actors, Daly [24] rightly states that professional groups, market-based actors, employers, and non-governmental organizations are associated with the growth of parenting support. As each of these groups has a vested interest in the provision of parenting support, and they were the object of the study, it was likely problematic for them to provide objective insights on the role of each actor without having implications for their respective work. Research directly with parents may give a more objective view of the role and value added by different key actors in promoting EBPS. When considering adjacent or encompassing policy domains, it is crucial to consider the use of conditional cash transfers alongside the provision of EBPS [24]. Cash transfers could be regarded as a form of parental support, and if parents, especially fathers as primary breadwinners, could receive cash assistance, this may increase their participation in EBPS. Although an EPBS combined with cash transfer has already been tested in Tanzania [30], policymakers and service providers did not raise it, as it is likely regarded as an expensive strategy.

Conversely, the authors noted factors not comprehensively addressed in Daly’s framework. These include the importance of sensitization and dissemination of information, the need to develop ongoing and targeted capacity, and the importance of addressing gender and social norms. Parents need to be informed and sensitized to the availability of EBPSs in a non-stigmatizing manner to increase demand. Simultaneously, staff need to be trained on core evidence-based concepts around the provision of parenting support in order to ensure both universal support (for all parents) and targeted support (for parents with more specific needs). Finally, and more overarchingly, according to policymakers and service providers, the roles of both men and women as involved caregivers would need to be addressed, as well as the role of children as active agents. While Daly’s framework remains a highly valuable tool for understanding and analyzing the provision of parenting support to prevent violence against children, these additional components could be included in future research.

### Implications for practice

The findings hold several implications for parenting practices in Tanzania [38]. Firstly, policymakers and service providers noted the need to disseminate and sensitize the policies to the local communities to drive the demand for parenting support. Laws and policies that support social equality (e.g., keeping children in school or supporting parents) are essential to changing norms and values that support violence against children [39]; however, uptake of EBPS will only occur if these policies and opportunities are communicated in a clear, comprehensive manner. Secondly, linked to communications and capacity development, informants noted that national efforts need to be put in place to ensure shared knowledge and understanding of EBPS as well as how they can be implemented based on a national parenting support strategy for Tanzania. Professionals and paraprofessionals (e.g., community health workers) can then be trained to implement the nationally endorsed EBPS in a coordinated manner at scale. Health research from Sub-Saharan Africa research suggests that laypersons who hold social capital (i.e., popular opinion leaders) could be trained to change prevention-related attitudes around the use of violence and accessing parenting support [40].

Thirdly, as noted by the informants, a very targeted approach is necessary to ensure that the support to parents aligns with the different realities in, for example, urban and rural settings. Programmes must be created or adapted using a participatory action research model with input from community stakeholders, including children, caregivers, educators, and religious/cultural leaders [40]. Associated with the targeted approach, policymakers and service providers raised the need to challenge the deeply entrenched systems, structures, and norms around gender (i.e., the power of men over women) as well as adultism (i.e., usually seen as the abuse of the power that adults have over children [41]). Policymakers and service providers themselves may have to clarify their own norms and values as they undertake this transformative work. Whereas social norms interventions are less costly than intensive parenting skills interventions [40], they may not be sufficient to change the individual behaviours of parents with regard to attending the parenting support classes and, therefore, should be considered alongside EBPS.

Lastly, funding remains a critical issue. In Tanzania, like in many low- and middle-income countries where capital is most likely coming from private donors, foundations, and high-income countries, it is essential that commercialized programmes are affordable, and that those initiatives are feasible to implement. This includes reliance on community workers and, possibly, the use of digital delivery.

### Strengths, limitations, and suggestions for future research

This study has several strengths and limitations. The main strength is that, for the first time, interviews were conducted with Tanzanian policymakers and service providers on the topic of parenting support (in Dodoma and Dar-es-Salaam, despite complex travel and interview arrangements). Additionally, this study extended Daly’s framework to a sub-Saharan African country and used Daly's framework, for the first time, to examine and organize policymakers’ and service providers’ perceptions. This approach enabled a better understanding of both the Tanzanian situation as well as how the framework can be used nationally and globally.

The main limitation is the small sample of informants due to the limited number of informants on this topic. The literature suggests that in qualitative research, the sample size should be large enough to allow encapsulation and understanding of new phenomena but small enough so that a thorough analysis of the data collected can be undertaken [42], which has been the case for this study. Linked to this issue, the study relied on government decision-makers and service providers to provide their views, therefore not considering parents' perspectives. Nonetheless, policymakers and service providers were generally also parents and provided both professional and personal perspectives. A further consideration is that violence against children and its reduction through parenting support is a sensitive and controversial topic, and participants may have given socially desirable responses. The issue of socially desirable responses tried to be resolved by having a national or international researcher. However, more research may be needed to understand the best approach to avoid socially desirable responses. Based on the findings of this study, the authors would encourage using Daly’s framework to understand enablers and challenges for country-level scale-up and potentially expand Daly’s framework to include missing elements that may emerge from national research contexts.

## Conclusion

In countries around the world, violence against children, and especially the use of violent discipline, remains a societal issue, and presently, only 67 out of 197 countries fully prohibit corporal punishment in the home and in schools [43]. Parenting support is a proven approach to curb the use of violent discipline [44], and Tanzania is seemingly aiming to scale-up EBPS to support parental well-being and decrease household violence. Our findings show that while many bridges between policy and practice have been built, significant challenges remain in scaling up parenting support in Tanzania. Our findings contribute to identifying factors that need to be addressed to close the gap between policies and implementation in the area of parenting support in Tanzania and may also be helpful for future research in countries worldwide.

## Supplementary Information

Supplementary Material 1.

## Data Availability

As the data collection was conducted jointly between the UU and NIMR; the recordings, transcriptions, and consent forms are saved on secured institutional servers UU (Yoda) and NIMR. The datasets used during the current study are available from the corresponding author upon reasonable request.
