# Supplementary figures and images for: Scaling up of parenting support to prevent violence against children in Tanzania: insights from policymakers and service providers
Source: Implement Sci Commun. 2025 Jan 13;6:8. doi: 10.1186/s43058-024-00684-8 (PMC11730138; doi:10.1186/s43058-024-00684-8)

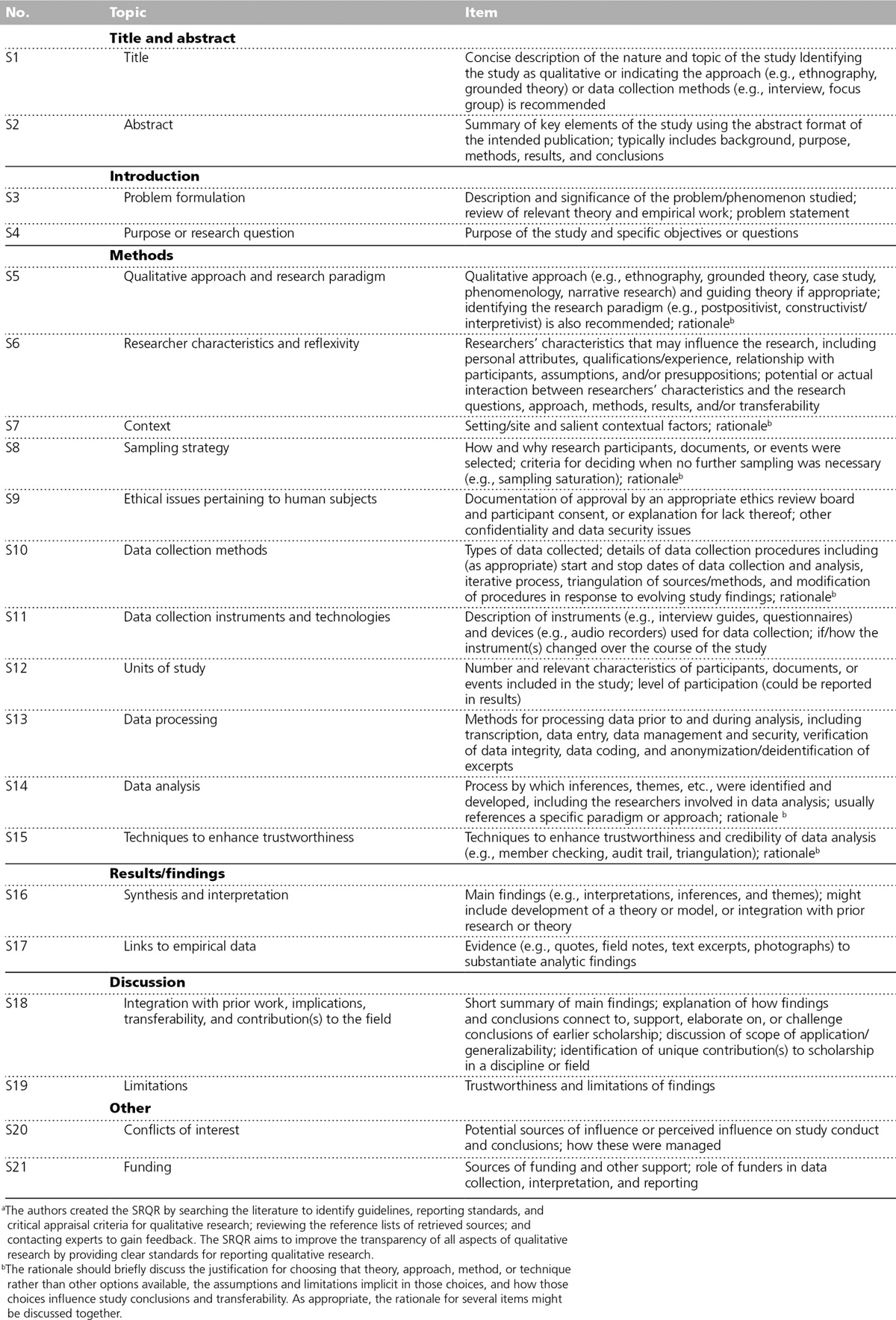

Supplement: Supplementary file 1 — Supplementary Material 1. [file 43058_2024_684_MOESM1_ESM.jpeg]
